# IT'S A VERY MANIPULATABLE TOOL: CHARACTERISTICS OF PAL CARD USE THAT IMPACT SUCCESSFUL IMPLEMENTATION

**DOI:** 10.1093/geroni/igac059.443

**Published:** 2022-12-20

**Authors:** Reese Moore, Megan Kelley, Miranda Kunkel, Kamryn Kasler, Julia Baker, Alexandra Heppner, Katherine Abbott, Kimberly Van Haitsma

**Affiliations:** Miami University, Oxford, Ohio, United States; Miami University, Oxford, Ohio, United States; Miami University, Oxford, Ohio, United States; Miami University, Oxford, Ohio, United States; Miami University, Oxford, Ohio, United States; Miami University, Oxford, Ohio, United States; Miami University, Oxford, Ohio, United States; Pennsylvania State University, University Park, Pennsylvania, United States

## Abstract

Understanding the barriers and facilitators of an intervention can inform implementation efforts. The purpose of this study was to understand the characteristics associated with the PAL Card intervention that led to successful implementation in nursing home (NH) settings. Qualitative telephone interviews were conducted with n=11 NH champions who completed the PAL Card QIP. Interviews were recorded, transcribed verbatim, and coded using the CFIR Intervention Characteristics domain in Dedoose. Three main themes regarding the intervention characteristics emerged including, relative advantage (i.e., advantage of using PAL Cards versus an alternate intervention), adaptability (i.e., how well the PAL Cards can be altered to meet community needs), and complexity (i.e., perceived difficulty of PAL Card usage). Participants voiced the simplicity and benefit of PAL Card implementation within their communities for both staff and residents alike. Implications for policy and practice will be discussed.

